# Splenic Rupture in an Elective Cesarean Section: A Possible Iatrogenic Event

**DOI:** 10.7759/cureus.48478

**Published:** 2023-11-07

**Authors:** Ioannis Korkontzelos, Gerasimia Kyrochristou, Stefanos Stefanou, Kyriaki Spyropoulou, George Mpourazanis, Daphne J Theodorou, Anna Papanikolaou, Pantelina-Danai Korkontzelou, Vasiliki E Georgakopoulou, Petros Papalexis

**Affiliations:** 1 Department of Obstetrics and Gynecology, Ioannina General Hospital “G. Chatzikosta”, Ioannina, GRC; 2 Department of Surgery, University Hospital of Ioannina, Ioannina, GRC; 3 Department of Surgery, Ioannina General Hospital “G. Chatzikosta”, Ioannina, GRC; 4 Department of Radiology, MRI-CT, Ioannina General Hospital “G. Chatzikosta”, Ioannina, GRC; 5 Faculty of Medicine, Medical University of Sofia, Sofia, BGR; 6 Department of Pulmonology, Laiko General Hospital, National and Kapodistrian University of Athens, Athens, GRC; 7 First Department of Pulmonology, Sismanogleio General Hospital, Athens, GRC; 8 Department of Medicine, Faculty of Health Sciences, Aristotle University of Thessaloniki, Thessaloniki, GRC; 9 Department of Endocrinology, First Department of Internal Medicine, Laiko General Hospital, National and Kapodistrian University of Athens, Athens, GRC

**Keywords:** iatrogenic event, spleen, splenic rupture, cesarean section, pregnancy

## Abstract

A rare case of an unexpected iatrogenic splenic rupture during a cesarean section is reported. The trauma was recognized early and treated conservatively without delay; thus, further complications were avoided. A 28-year-old woman with a history of previous cesarean sections was submitted for an elective cesarean section. Intra-operatively, minor bleeding from the left abdomen was noted and eventually assigned to an inferior pole splenic trauma treated conservatively without splenectomy. Although unclear, the injury was considered iatrogenic, probably due to the abdominal pressure for fetal delivery and the anatomy of the splenocolic ligament. This case highlights the clinical suspicion that is required despite routine surgical procedures for the early diagnosis of an unexpected splenic rupture when minor bleeding occurs intra-operatively from the upper abdomen. Early recognition and prompt treatment are of paramount importance for the safety of the fetus and the patient.

## Introduction

In general, hemorrhage in pregnancy is the leading cause of maternal and fetal morbidity and mortality worldwide, with medical, obstetrical, and surgical etiologies. Some authors consider that pregnancy per se is a risk factor for splenic rupture, explained by pregnancies’ hypervolemia and alterations of the abdominal organs [1]. This potentially lethal injury could occur with any degree of trauma to a normal spleen or a minor trauma to a diseased spleen and has been reported in patients with or without predisposing factors [2,3]. In some cases, the mechanism of injury is unclear and multifactorial [4,5].

Splenic rupture is labeled as spontaneous when it is not associated with antecedent trauma, systemic disease, or gross pathology and the parenchyma, the vasculature, and the capsule are normal [2]. Rupture of a splenic artery aneurysm is another entity but is also a rare and life-threatening complication in pregnancy, with an incidence of 0.01%-10.4% [6].

Iatrogenic splenic rupture injury is defined as any involuntary damage caused to the spleen during an operation or a medical intervention [7]. Intraoperative bleeding originating from the spleen is an extremely rare complication of cesarean section but could significantly increase the risk of maternal morbidity and mortality during or after childbirth [8]. The reported maternal mortality from splenic rupture ranges between 0% and 45%, with a 47-82% risk of fetal wastage [1,5].

## Case presentation

A 28-year-old woman, gravida 3, para 1, was admitted for an elective cesarean section at 38+1 weeks of gestation. Her obstetrical history included a previous cesarean section and a pregnancy termination due to a fetal cardiac abnormality. The medical history was insignificant, without splenomegaly and a normal body mass index (24.4 kg/m2). Under general anesthesia, a Pfannenstiel incision was performed. After a low transverse uterine incision and rupture of the membranes, abnormal fetal presentation was noted, and standard maneuvers to establish vertex presentation followed. A healthy female infant weighing 3100gr was delivered on time with Apgar scores of 8 and 10 in the 1st and 5th minutes, respectively. Routine closure of the uterine wall was accomplished with minimal blood loss. Intra-operatively, during the final control of the adnexas, the appendix, and the abdominal cavity, barely underlined but continuous minor bleeding coming from the upper left abdomen was ascertained. The general surgeon was called, and after evaluation, he approached the left upper quadrant with a Kocher incision, and the bleeding site was revealed. A splenic trauma had occurred at the inferior pole of the spleen, at the point where the splenocolic ligament is attached, which was noted to be extremely thin. The conservative technique of packing, topical surgical hemostatic agents, and non-absorbable sutures was enough to control the bleeding, and splenectomy was prevented. The estimated blood loss from the splenic injury was approximately 80 ml. The patient remained hemodynamically stable during surgery, and the postoperative period was uneventful.

**Figure 1:**
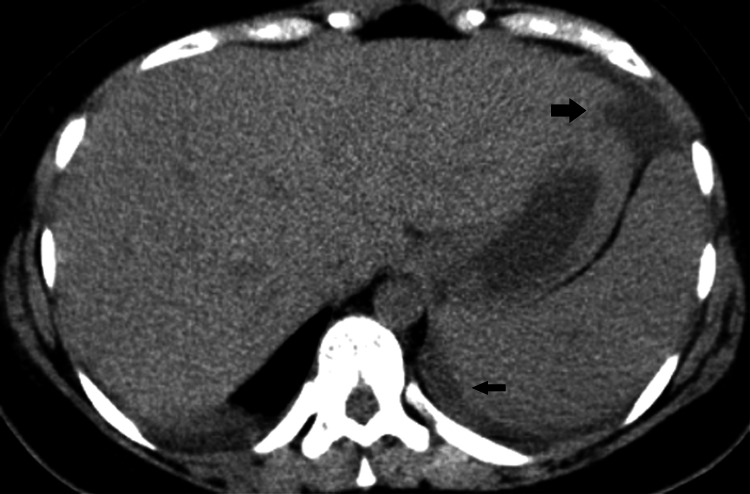
Axial non-enhanced computed tomography image Low-density fluid (arrow) adjacent to the spleen due to subcapsular hematoma. There is free fluid in the greater sac (thick arrow).

**Figure 2:**
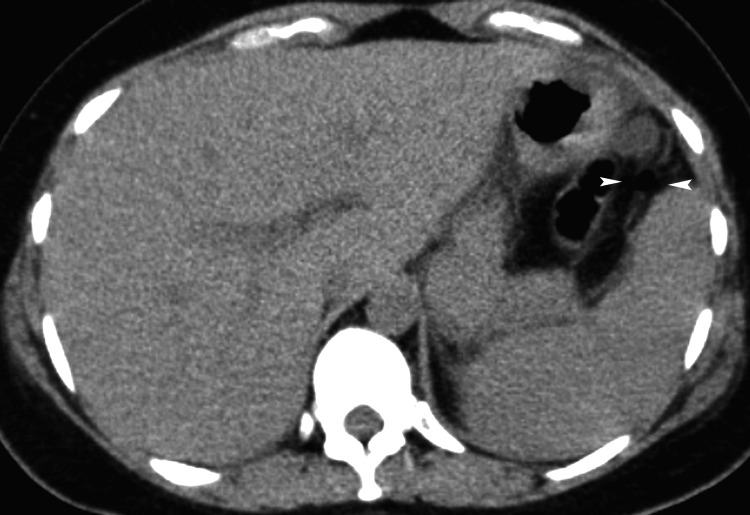
Axial computed tomography (CT) image CT image shows free air (arrowheads) in the peritoneum.

No pathological findings were indicated by the computed tomography (CT) scan after the operation (Figure 1, 2).

The mechanism of rupture was discussed but remained unclear, attributed most probably to the abdominal pressure for fetal delivery and the anatomy of the splenocolic ligament. The patient was discharged on the fourth day in good condition, and follow-up after forty days was normal. The laboratory results concerning hemodynamic stability before and after the operation are listed in Table 1.

**Table 1:** Laboratory diagnostic follow up of the hemodynamic status of the patient before and after the operation INR: international normalized ratio; PLT: platelets

Parameter	Day 0 (admission & cesarean section)	Day 1	Follow up (40 days)	Reference range
Hematocrit	32%	29.2%	37%	36-42%
Hemoglobin	10 gr/dl	9.7 gr/dl	11.9 gr/dl	12-14 gr/dl
PLT	199.000 K/μL	176.000 K/μL	209.000 K/μL	140-400 K/μL
INR	1.02	_	_	0.9-1.20

## Discussion

If the spleen suffers an unintentional injury or rupture in an elective or urgent operation or medical intervention, it is defined as an “iatrogenic splenic rupture". The exact incidence of iatrogenic splenic trauma is hard to define since many cases of intraoperative complications that are treated conservatively are not finally described. This leads to an underestimation of this medical condition, which constitutes an indication for up to 40% of all splenectomies [7].

Overtraction of the ligaments attached to the spleen (especially the spleno-colic ligament) is the main mechanism of splenic rupture. Anatomical variations of the splenic hilum and the splenic artery branches may also contribute to incidental injury. Another cause could be the mobilization of the left colonic flexure and the traction at any portion of the greater omentum due to its close attachments with the splenic capsule. A previous medical history of surgical procedures may contribute to the presence of adhesions and constitute an independent risk factor for injury [9].

The physiological changes following pregnancy may predispose to splenic injury. At first, increased circulation of estrogen and progesterone may increase the size of the spleen and splenic vasculature. The spleen position may also alter or lead to increased mobility. In general, splenic rupture occurs more often in women with advanced maternal age, in multiparous women, in multiple gestations, and in the third trimester of pregnancy, although cases in the peripartum and postpartum periods have also been reported. In addition, previous abdominal operations (presence of adhesions, emergent laparotomy, peripheral vascular disease, and obesity) could be present [1,2].

Spontaneous peripartum splenic rupture has been reported in patients with hemolysis syndrome, elevated liver enzymes, a low platelet count, preeclampsia, and splenic ectopic pregnancy. In the same period, increased circulation of blood volume or administration of fluid and blood products could engorge the spleen, making the organ more susceptible to injury. The most frequent clinical symptom is pain on the left side spreading up to the left shoulder. Interestingly, spontaneous rupture is similar in vaginal and cesarean deliveries [1,3].

Splenic rupture occurring during a cesarean section is quite rare. Trauma could occur from the abdominal incision, the insertion of packs, maneuvers for the neonatal expulsion associated with forceful pulsing on the upper abdomen and the uterine fundus, the use of retractors and sharp instruments, or even while removing clots from the paracolic gutters [2,3]. If the bleeding is not seen intra-operatively, the splenic capsule may remain intact for hours, but eventually it will rupture because of the increased subcapsular pressure, with catastrophic consequences.

However, Sakhel et al. [2] reported two cases in which the etiology of splenic rupture remained unclear. In the first case, the patient was pre-eclamptic and presented tonic-clonic seizures, but the injury was attributed most probably to a short splenic pedicle. Furthermore, in both patients, rapid plasma expansion with blood products and other volume expanders was given. This might have resulted in a rapid volume increase within the spleen, and the authors consider that this could be another predisposing factor for its rupture.

Physical examination and laboratory tests are crucial in establishing a diagnosis. Abdominal ultrasound is useful in the quick diagnosis of intraperitoneal fluid loss or splenic hematoma and could be performed in the emergency unit or the theater. Additionally, a CT scan could also be used [10].

Splenic rupture is most common in the peripartum and postpartum periods and appears to be difficult to diagnose. Common entities like postpartum pain after a cesarean section, intra-abdominal uterine bleeding, uterine rupture, or injury of the viscus present with similar findings. If shock is established, the differential diagnosis could be amniotic fluid embolism, pulmonary embolism, cardiogenic shock, intravascular coagulopathy, or septic shock [2].

The management of splenic rupture may be conservative in milder lesions (packing, topical surgical hemostatic agents, argon beam coagulation, bio-absorbable hemostatic agents, mesh utilization, selective vessel ligation, suture repair, splenorrhaphy, or segmental section) or aggressive, ending in total splenectomy (open, laparoscopic, or robotic) [9,11].

Splenic artery angiography followed by embolization has also been described with a success rate of 85%; however, its role in the final management is unclear [12]. The therapeutic decision is still a matter of scientific debate and may be based on the patient’s condition and hemodynamic stability, the wound extension, or the surgeon’s expertise. Until now, almost 85% of splenic injuries have been handled by total splenectomy; however, it should only be performed when conservative management fails to control active bleeding. Spleen-sparing techniques not only preserve the organ’s immune function but also reduce the length of hospitalization and the need to repeat surgery [9,11,13].

Intraoperative splenic rupture during gynecological or obstetrical procedures is extremely rare but may cause significant morbidity. Bladder, ureteral, and gastrointestinal injuries are more commonly described. The need to extend the length of the initial Pfannenstiel incision is always possible when a difficult cesarean section is performed. In cases of known fetal malpresentation, vacuum-assisted delivery could be performed to avoid excessive abdominal pressure, and the obstetrician should not hesitate to call the general surgeon when necessary [14]. Furthermore, an immediate large rupture is easy to recognize, but in small traumas, blood loss is minimal, and delayed manifestations could be catastrophic. Accurate diagnosis and immediate intervention are crucial to avoid long-term thrombotic, immunologic, and infectious risks following splenectomy [15].

## Conclusions

This is believed to be the first report of iatrogenic splenic rupture during a cesarean section with maintenance of the spleen and a favorable outcome. This case highlights the clinical suspicion required when unexpected minor but continuous bleeding originating from the upper abdomen occurs, and this hemorrhage must not be ignored. Our patient recovered very well, and splenectomy was avoided because the bleeding was recognized promptly, she remained hemodynamically stable, and the injury was small.
